# Validation of non‐muscle‐invasive bladder cancer risk stratification updated in the 2021 European Association of Urology guidelines

**DOI:** 10.1002/bco2.305

**Published:** 2023-11-03

**Authors:** Makito Miyake, Hiroshi Kitamura, Nobutaka Nishimura, Tatsuki Miyamoto, Tomonori Nakahama, Tomomi Fujii, Hiroaki Matsumoto, Hideyasu Matsuyama, Masaya Yonemori, Hideki Enokida, Rikiya Taoka, Takashi Kobayashi, Takahiro Kojima, Yoshiyuki Matsui, Naotaka Nishiyama, Hiroyuki Nishiyama, Kiyohide Fujimoto, Nishinihon Uro‐oncology Extensive Collaboration group and the Japanese Urological Oncology Group

**Affiliations:** ^1^ Department of Urology Nara Medical University Kashihara Nara Japan; ^2^ Department of Urology, Faculty of Medicine University of Toyama Toyama Japan; ^3^ Department of Diagnostic Pathology Nara Medical University Kashihara Nara Japan; ^4^ Department of Urology, Graduate School of Medicine Yamaguchi University Ube Yamaguchi Japan; ^5^ Department of Urology JA Yamaguchi Kouseiren Nagato General Hospital Nagato Japan; ^6^ Department of Urology, Graduate School of Medical and Dental Sciences Kagoshima University Kagoshima Japan; ^7^ Department of Urology, Faculty of Medicine Kagawa University Takamatsu Kagawa Japan; ^8^ Department of Urology Kyoto University Graduate School of Medicine Kyoto Japan; ^9^ Department of Urology Aichi Cancer Center Nagoya Aichi Japan; ^10^ Department of Urology National Cancer Center Hospital Tokyo Japan; ^11^ Department of Urology, Faculty of Medicine University of Tsukuba Tsukuba Ibaraki Japan

**Keywords:** prediction, prognosis, progression, recurrence, risk, urinary bladder neoplasms

## Abstract

**Objective:**

The objective of this study is to validate the predictive ability of the 2021 European Association of Urology (EAU) risk model compared to that of existing risk models, including the 2019 EAU model and risk scoring tables of the European Organization for Research and Treatment of Cancer, Club Urologico Espanol de Tratamiento Oncologico, and Japanese Nishinihon Uro‐oncology Extensive Collaboration Group.

**Patients and methods:**

This retrospective multi‐institutional database study included two cohorts—3024 patients receiving intravesical bacillus Calmette–Guerin (BCG) treatment (BCG cohort) and 789 patients not receiving BCG treatment (non‐BCG cohort). The Kaplan–Meier estimate and log‐rank test were used to visualize and compare oncological survival outcomes after transurethral surgery among the risk groups. Harrell's concordance index (C‐index) was used to evaluate the predictive ability of the models.

**Results:**

We observed a risk shift from the 2019 EAU risk grouping to the 2021 EAU risk grouping in a substantial number of patients. For progression, the C‐index of the 2021 EAU model was significantly higher than that of the 2019 EAU model in both the BCG (0.617 vs. 0.572; *P* = 0.011) and non‐BCG (0.718 vs. 0.560; *P* < 0.001) cohorts. According to the 2021 EAU model, 731 (24%) and 130 (16%) patients in the BCG and non‐BCG cohorts, respectively, were considered to have a very high risk. Survival analysis showed no significant differences among the five very high‐risk subgroups in both cohorts. A major limitation was potential selection bias owing to the retrospective nature of this study.

**Conclusions:**

The updated 2021 EAU model showed better stratification than the three existing risk models, especially for progression, in both cohorts, determining the most appropriate postoperative treatment and identifying patients requiring intensified surveillance or early cystectomy.

## 1 INTRODUCTION

Bladder cancer is the 10th most commonly diagnosed malignancy worldwide, with 573 000 new cases and 213 000 deaths reported.^1^ In the European Union, the age‐standardized incidence rate is 27 and 6 per 100 000 individuals among men and women, respectively. Among newly diagnosed patients, most (70–80%) have non‐muscle‐invasive bladder cancer (NMIBC) forms of the disease, which include Ta, T1 and Tis classifications. Despite having a low lifetime risk of death, these NMIBC forms are characterized by high rates of intravesical recurrence and progression to muscle‐invasive bladder cancer.^2^, ^3^ The heterogeneous clinical and biological behaviours of NMIBC require tailored treatment recommendations, such as intravesical chemotherapy and intravesical bacillus Calmette–Guerin (BCG) treatment, as well as follow‐up regimens involving hospital visits and invasive cystoscopy examinations.

The 2019 European Association of Urology (EAU) guidelines recommend stratification of patients with NMIBC into three risk groups based on the World Health Organization (WHO) 2004/2016 classification system, which included low‐, intermediate‐ and high‐risk subgroups, with the latter including the highest‐risk subgroup.^4^ In 2021, the EAU‐NMIBC Guidelines Panel Study demonstrated that a four‐tier combination (low‐grade [LG]/G1, LG/G2, high‐grade [HG]/G2 and HG/G3) of both the WHO 1973 and WHO 2004/2016 grading systems was superior to a two‐subgroup (LG and HG) grading systems for predicting progression risk.^5^ Consequently, the 2021 updated EAU guidelines proposed a four‐group risk stratification model utilizing the widely available clinicopathological features from both tumour grading systems.^6^, ^7^

An external validation study including 529 patients treated with induction BCG (iBCG) with or without maintenance BCG (mBCG) demonstrated that the 2021 EAU risk model overestimated progression risks and reduced the discriminative ability in the validation cohort and despite a successful stratification of progression risks, treatment with BCG reduced the discriminative ability.^8^ Another external validation study conducted in Korea validated the applicability of the 2021 EAU risk stratification for risk of progression.^9^ However, the study did not investigate the comparative superiority or inferiority of the model to other existing risk models or explore the association between the risk stratification model and bladder recurrence risk or cancer‐specific death.^8^, ^9^ Therefore, this multi‐institutional validation study aimed to validate the proposed risk model through comparisons with the previous 2019 EAU model and risk tables of the European Organization for Research and Treatment of Cancer (EORTC),^10^ Club Urologico Espanol de Tratamiento Oncologico (CUETO)^11^ and Japanese NIshinihon uro‐onCology Extensive collaboration group (J‐NICE)^12^ with two large‐scale cohorts of patients treated with and without intravesical BCG.

## 2 PATIENTS AND METHODS

### 2.1 Two validation cohorts of NMIBC

**FIGURE 1:**
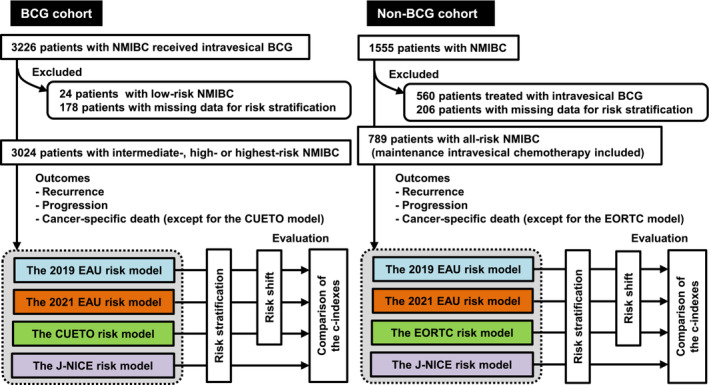
Flow chart for the patient's cohort data sets and schematic design of this study. Two independent datasets are used for external validation: the BCG cohort receiving intravesical BCG treatment and the non‐BCG cohort that did not receive BCG treatment. From the original datasets, the cohorts excluded patients with critically missing data for risk stratification in each model. BCG, bacillus Calmette‐Guerin; CUETO, Club Urologico Espanol de Tratamiento Oncologico; EAU, European Association of Urology; EORTC, European Organization for Research and Treatment of Cancer; J‐NICE, Japanese Nishinihon Uro‐oncology Extensive Collaboration Group; NMIBC, non‐muscle‐invasive bladder cancer.

This multicentre study was conducted in collaboration with researchers across Japan and was approved by the institutional review boards (reference ID: 1487, 2217, H29‐045 and 21‐090) of the J‐NICE and Japanese Urological Oncology Group frameworks. Informed consent was obtained from the participants or bereaved families through posters and/or websites using the opt‐out method.^13^ Figure 1 illustrates the flowchart of the patient enrolment and the schematic design of this study.

This validation study included two cohorts—the BCG and non‐BCG cohorts. The BCG cohort comprised 3024 patients diagnosed with NMIBC between 2000 and 2018 who received intravesical BCG treatment. The database of the BCG cohort was previously developed in the Japanese Urological Oncology Group framework with the intention of a retrospective comprehensive analysis of patients treated with intravesical BCG. The dose and schedule for iBCG and mBCG were inconsistent and determined at the physician's discretion and patient preferences. The iBCG schedule included weekly administration of ImmuCyst (81 mg of Connaught strain) or Immunobladder (80 mg of Tokyo‐172 strain) for six to eight consecutive weeks with or without administration of mBCG. mBCG was administered weekly for 3 weeks at 3, 6, 12, 18, 24, 30 and 36 months after iBCG initiation.^2^ The J‐NICE previously developed a large‐scale database of 1555 patients diagnosed with NMIBC between 1980 and 2016.^12^ Of these patients, 560 patients treated with intravesical BCG and 206 patients with missing data for risk stratification were excluded; hence, the non‐BCG cohort included 789 eligible patients who did not receive intravesical BCG treatment. The cohort did not include patients with prostate carcinoma in situ (CIS) but without bladder urothelial carcinoma.

Clinicopathological data obtained from each hospital included information regarding age, sex, T categories and tumour grades based on both the WHO 1973 and WHO 2004/2016 systems of transurethral resection of bladder tumour (TURBT) specimens, tumour size, tumour multifocality, presence of concomitant CIS, presence of variant histology, prior recurrence history of NMIBC, immediate postoperative instillation of chemotherapy, adjuvant intravesical therapy and clinical outcomes. A central pathology review was conducted if TURBT was performed before 1998.

### 2.2 Risk stratification with different models

Clinicopathological factors were used for risk grouping based on the 2019 EAU, 2021 EAU, CUETO, EORTC and J‐NICE stratification models. The CUETO model, designed for patients treated with intravesical BCG,^11^ was specifically applied to the BCG cohort. Conversely, the EORTC model, which was devised from a dataset containing a low number of patients treated with BCG,^10^ was applied to the non‐BCG cohort. The J‐NICE risk‐scoring model was designed to predict the risk of recurrence, progression and cancer‐specific death.^12^ The high‐risk group of the 2019 EAU risk stratification was defined as patients who had at least one of the high‐risk factors but did not have any of the highest risk factors. Tables S1 and S2 present the composition and scoring tables of the five stratification models. In the EORTC and CUETO models, four‐tier stratification into the low‐, intermediate‐, high‐ and highest‐risk groups was applied according to previously reported calculated scores.^10^, ^11^ Papillary urothelial neoplasia with low malignant potential and LG tumours were combined into one LG category because of their similar prognoses, as previously demonstrated.^14^

### 2.3 Follow‐up and endpoints

Patients were monitored according to the clinical practice guidelines during the follow‐up period. This study evaluated three outcomes—recurrence‐free survival (RFS), progression‐free survival (PFS) and cancer‐specific survival (CSS). Recurrence was defined as a pathologically confirmed intravesical recurrent tumour including LG/G1 disease, but excluding residual disease at restaging TUR. Progression was defined as invasion of the muscularis propria (≥T2) or development of lymph nodes and/or distant metastasis. Primary Ta tumours to subsequent T1 tumours or primary LG tumours to subsequent HG tumours were not considered as progression. Cancer‐specific death was defined as death attributable to urothelial carcinoma.

### 2.4 Statistical analysis and software

Statistical analyses and data visualization were performed using PRISM software version 9 (GraphPad Software, Inc., San Diego, CA) and R version 4.2.2 (packages: riverplot, survival and compareC; https://www.r-project.org/). Statistical significance was defined at *P* values < 0.05. Sankey diagrams were used to present a visual representation of the risk shift from the 2019 EAU model to the 2021 EAU model or the CUETO/EORTC model for the BCG/non‐BCG cohorts. The Kaplan–Meier estimate and log‐rank test were used to visualize and compare survival outcomes after TURBT among the risk groups. Harrell's concordance index (C‐index), which ranges from 0 to 1 (with 1 indicating perfect concordance), was used to evaluate the prognostic accuracy of the risk‐stratification models for the three outcomes for right‐censored event times. The predictive ability of these risk models was compared based on their C‐indices.

## 3 RESULTS

### 3.1 Patient characteristics and risk grouping

**FIGURE 2:**
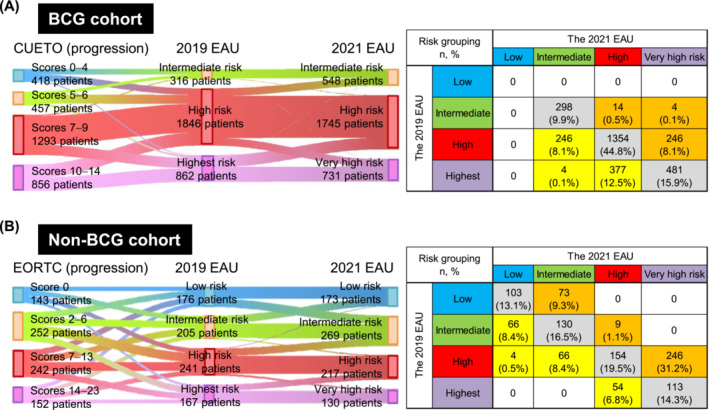
Shift of the risk grouping from the 2019 EAU model to the 2021 EAU model in the BCG (A) and non‐BCG (B) cohorts. Sankey diagrams (left panels) connecting the risk grouping from the CUETO or EORTC models (based on the progression score) to the 2019 EAU model and from the 2019 EAU model to the 2021 EAU model. Each vertical bar represents stratified risks: blue, low risk; yellow, intermediate risk; red, high risk; purple, highest risk; or very high risk. Tabulation of risk grouping by the 2019 EAU model and the 2021 risk model (right panels) shows patients with a reduced risk (yellow), an increased risk (orange) and a risk agreement (grey). CUETO, Club Urologico Espanol de Tratamiento Oncologico; EAU, European Association of Urology; EORTC, European Organization for Research and Treatment of Cancer.

The clinicopathological characteristics of the BCG and non‐BCG cohorts are presented in Tables S3 and S4, respectively. Using the 2021 EAU model, the BCG cohort was stratified into the intermediate‐ (*n* = 548; 18%), high‐ (*n* = 1745; 58%) and very high‐risk (*n* = 731; 24%) groups, and the non‐BCG cohort was stratified into the low‐ (*n* = 173; 22%), intermediate‐ (*n* = 269; 34%), high‐ (*n* = 217; 28%) and very high‐risk (*n* = 130; 16%) groups. A substantial proportion of patients was shifted between risk categories when comparing the risk grouping of the 2019 EAU with that of the 2021 EAU (Figure 2). Of the 3024 patients in the BCG cohort, 627 (21%) showed a decrease in risk, whereas 264 (8.7%) showed an increase in risk. Of the 789 patients in the non‐BCG cohort, 190 (24%) showed a decrease in risk, while 328 (42%) showed an increase in risk.

### 3.2 Risk stratification for recurrence, progression, and cancer‐related deaths

**FIGURE 3:**
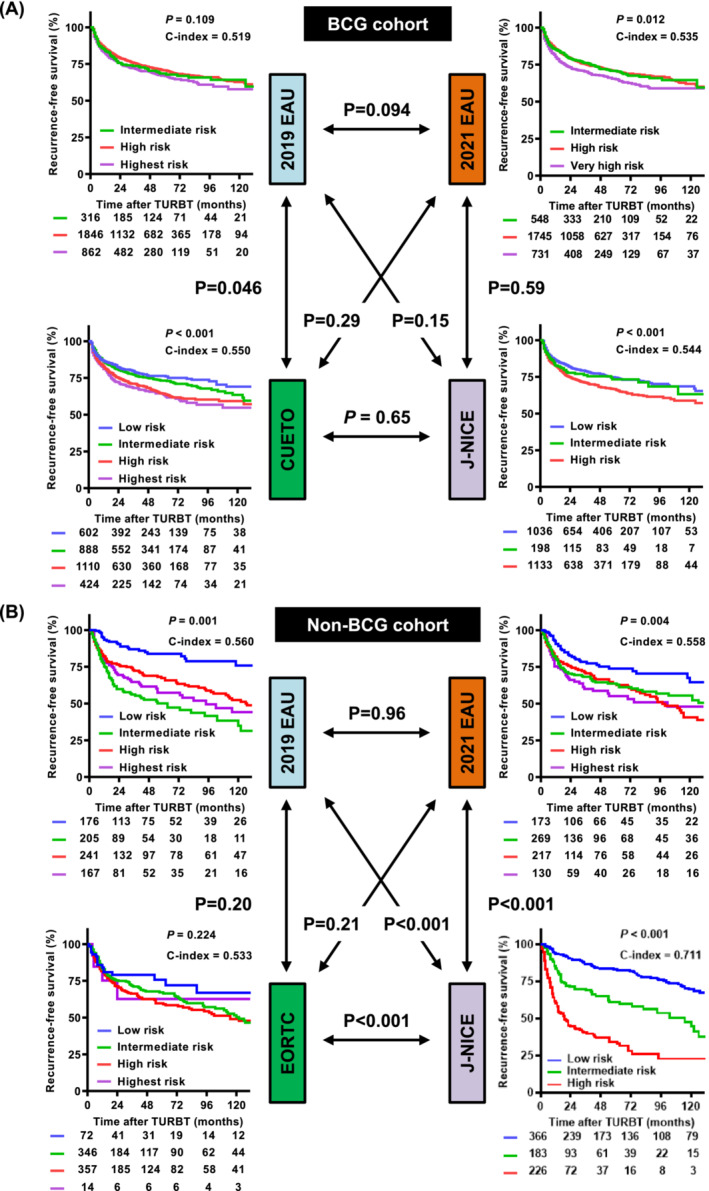
Recurrence‐free survival curves and comparison of predictive ability among risk models in the BCG (A) and non‐BCG (B) cohorts. Survival curves stratified by each stratification model were compared using the log‐rank test, and the predictive ability was evaluated using the C‐index. The overall C‐index values for the prediction of progression were compared using the CompareC package in the R software. The *p* values indicated by the double arrows represent the differences between C‐indices. BCG, bacillus Calmette‐Guerin; CUETO, Club Urologico Espanol de Tratamiento Oncologico; EAU, European Association of Urology; EORTC, European Organization for Research and Treatment of Cancer; J‐NICE, Japanese Nishinihon Uro‐oncology Extensive Collaboration Group; NMIBC, non‐muscle‐invasive bladder cancer.

**FIGURE 4:**
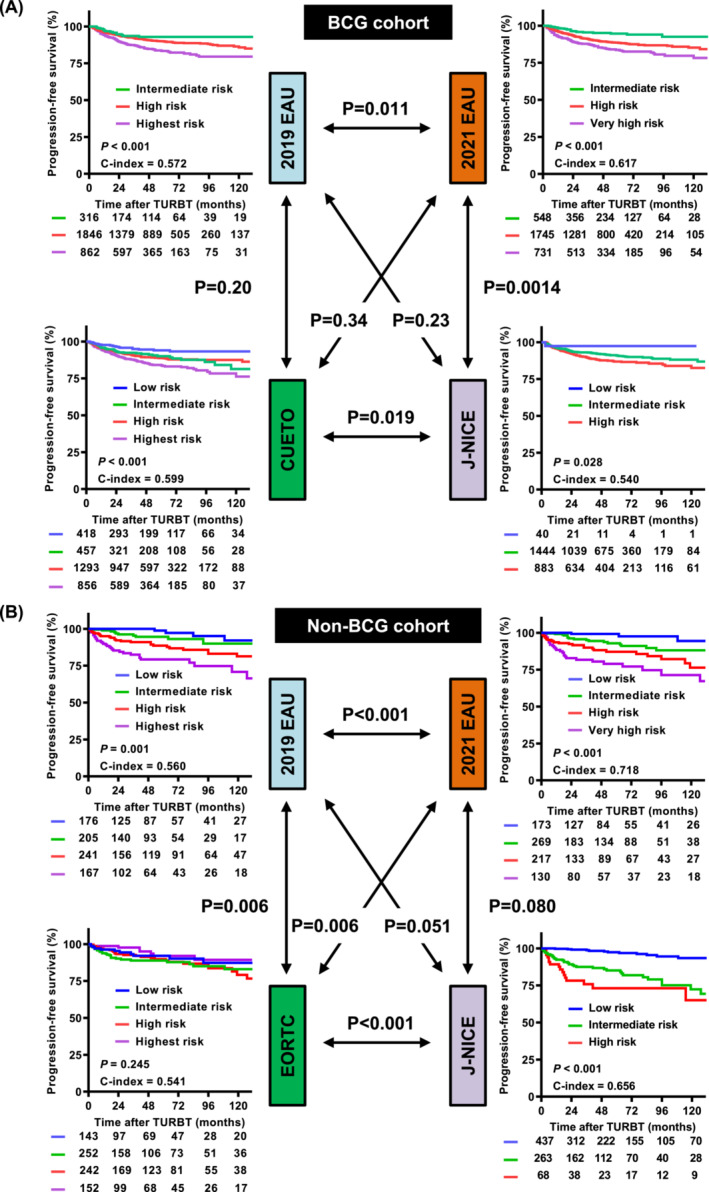
Progression‐free survival curves and comparison of predictive ability among risk models in the BCG (A) and non‐BCG (B) cohorts. Survival curves stratified by each stratification model were compared using the log‐rank test, and the predictive ability was evaluated using the C‐index. The overall C‐index values for the prediction of progression were compared using the CompareC package in the R software. The *p* values indicated by the double arrows represent the differences between C‐indices. BCG, bacillus Calmette‐Guerin; CUETO, Club Urologico Espanol de Tratamiento Oncologico; EAU, European Association of Urology; EORTC, European Organization for Research and Treatment of Cancer; J‐NICE, Japanese Nishinihon Uro‐oncology Extensive Collaboration Group; NMIBC, non‐muscle‐invasive bladder cancer.

The median follow‐up duration after TURBT was 48.0 (interquartile range [IQR], 26.0–75.0) months in the BCG cohort and 62.7 (IQR, 18.0–87.0) months in the non‐BCG cohort. Tables 1 and 2 show the event‐free survival rates at 1, 2, 5 and 10 years and their 95% confidence intervals (CIs) for patients stratified into the BCG and non‐BCG cohorts. Figures 3, 4, and S1 show comparisons of event‐free survival and C‐indices according to the risk stratification for recurrence, progression and cancer death, respectively. The progression analysis of our cohorts demonstrated consistent results, indicating that the C‐index of the 2021 EAU model was significantly higher than that of the 2019 EAU model in the BCG (0.617 vs. 0.572; *P* = 0.011) and non‐BCG (0.718 vs. 0.560; *P* < 0.001) cohorts. In the non‐BCG cohort, the intermediate‐risk group stratified using the 2019 EAU model had the highest recurrence risk, whereas that stratified using the 2021 EAU model had a low to very high recurrence risk (Table 3 and Figure S2). There was no difference in the predictive ability for CSS between the 2019 EAU, 2021 EAU and J‐NICE models (Figure S1).

**TABLE 1:** Estimated survival probabilities at 1, 2, 5 and 10 years in patients with NMIBC stratified by the risk models in the BCG cohort patients.

Risk group	Recurrence‐free survival % years (95% CI)				Progression‐free survival % years (95% CI)				Cancer‐specific survival % years (95% CI)			
	1 year	2 years	5 years	10 years	1 year	2 years	5 years	10 years	1 year	2 year	5 years	10 years
The 2019 EAU risk model												
Intermediate risk	84 (80–88)	77 (71–81)	69 (64–75)	65 (59–72)	97 (94–99)	95 (91–97)	93 (89–96)	93 (89–96)	99 (98–100)	99 (97–100)	96 (93–98)	95 (91–97)
High risk	85 (84–87)	79 (77–81)	71 (68–73)	63 (59–66)	96 (95–97)	94 (93–95)	90 (88–91)	86 (83–88)	99 (99–100)	99 (98–99)	96 (94–97)	92 (89–94)
Highest risk	82 (79–85)	76 (73–79)	67 (63–70)	58 (51–64)	94 (92–96)	90 (87–92)	83 (80–86)	80 (75–83)	99 (99–100)	99 (98–99)	94 (92–96)	87 (82–91)
The 2021 EAU risk model												
Intermediate risk	86 (82–89)	80 (75–83)	72 (67–77)	66 (60–72)	98 (96–99)	96 (94–98)	94 (91–97)	94 (92–97)	99 (98–100)	99 (98–100)	97 (95–99)	97 (93–98)
High risk	86 (84–87)	79 (77–81)	71 (68–73)	62 (58–65)	96 (95–97)	93 (92–94)	88 (87–90)	85 (83–87)	99 (99–100)	99 (98–99)	95 (94–96)	91 (88–93)
Very high risk	77 (73–80)	70 (66–74)	63 (58–67)	58 (53–64)	93 (90–95)	89 (86–91)	84 (80–87)	85 (83–88)	99 (98–100)	99 (97–99)	94 (92–96)	88 (82–93)
The CUETO model												
Low risk (scores 0–4)	87 (84–89)	83 (79–85)	77 (73–80)	70 (64–75)	98 (96–99)	96 (94–98)	94 (91–96)	94 (90–96)	‐	‐	‐	‐
Intermediate risk (scores 5–6)	86 (84–88)	80 (77–83)	73 (70–76)	63 (57–69)	96 (93–97)	93 (90–95)	90 (86–93)	81 (72–88)	‐	‐	‐	‐
High risk (scores 7–9)	83 (80–85)	75 (72–78)	65 (61–68)	59 (55–64)	96 (95–97)	93 (91–94)	89 (87–91)	88 (85–90)	‐	‐	‐	‐
Highest risk (scores 10–16)	81 (77–85)	72 (67–76)	64 (58–69)	55 (47–62)	95 (93–96)	90 (88–92)	84 (81–87)	76 (69–82)	‐	‐	‐	‐
The J‐NICE model (recurrence/progression/cancer‐specific death)												
Intermediate (scores 5–6/3–9/4)	83 (77–88)	79 (72–84)	74 (67–80)	65 (50–76)	96 (95–97)	94 (92–95)	91 (90–93)	88 (85–91)	99 (99–100)	99 (98–100)	96 (95–98)	94 (92–96)
High (scores 7–16/10–19/5)	83 (80–85)	75 (72–77)	67 (63–70)	59 (54–64)	96 (94–97)	92 (90–94)	87 (84–89)	83 (78–87)	99 (99–100)	98 (97–99)	94 (92–95)	86 (81–90)

Abbreviations: BCG, bacillus Calmette‐Guerin; CI, confidence interval; CUETO, Club Urologico Espanol de Tratamiento Oncologico; EAU, European Association of Urology; J‐NICE, Japanese NIshinihon uro‐onCology Extensive collaboration group; NMIBC, non‐muscle invasive bladder cancer.

**TABLE 2:** Estimated survival probabilities at 1, 2, 5 and 10 years in patients with NMIBC stratified by the risk models in the non‐BCG cohort patients.

Risk group	Recurrence‐free survival% years (95% CI)				Progression‐free survival% years (95% CI)				Cancer‐specific survival% years (95% CI)			
	1 year	2 years	5 years	10 years	1 year	2 years	5 years	10 years	1 year	2 years	5 years	10 years
The 2019 EAU risk model												
Low risk	93 (88–96)	90 (84–94)	84 (76–89)	76 (65–84)	‐	‐	99 (91–100)	92 (79–97)	‐	‐	‐	97 (79–99)
Intermediate risk	75 (68–81)	60 (52–67)	50 (42–58)	35 (23–47)	99 (96–100)	96 (92–98)	95 (89–97)	90 (79–95)	‐	‐	98 (93–100)	98 (93–99)
High risk	82 (76–87)	76 (70–81)	67 (60–74)	52 (44–60)	96 (91–97)	92 (88–95)	89 (83–93)	81 (73–87)	99 (96–100)	99 (96–100)	97 (92–99)	94 (88–97)
Highest risk	80 (72–85)	69 (61–76)	57 (48–66)	44 (32–56)	90 (84–94)	85 (78–90)	79 (71–85)	71 (58–81)	99 (95–100)	95 (89–98)	91 (84–95)	89 (81–94)
The 2021 EAU risk model												
Low risk	91 (85–94)	83 (75–88)	74 (65–81)	65 (52–75)	‐	99 (95–100)	99 (95–100)	95 (81–98)	‐	‐	99 (94–100)	96 (80–99)
Intermediate risk	81 (75–85)	71 (64–76)	64 (57–70)	56 (47–63)	99 (97–100)	96 (93–98)	93 (88–96)	88 (81–93)	‐	‐	99 (95–100)	99 (95–100)
High risk	82 (76–87)	75 (68–81)	64 (55–71)	41 (31–51)	94 (89–96)	92 (88–95)	87 (81–92)	76 (64–85)	99 (96–100)	98 (95–99)	96 (90–98)	94 (88–97)
Very high risk	75 (66–82)	66 (56–73)	55 (44–65)	48 (36–59)	88 (82–93)	83 (75–89)	79 (70–86)	71 (59–81)	98 (93–100)	94 (88–98)	91 (82–95)	85 (73–91)
The EORTC model (recurrence/progression)												
Low risk (scores 0/0)	84 (73–91)	79 (67–87)	76 (62–85)	67 (49–80)	96 (91–98)	95 (90–98)	92 (85–96)	87 (77–93)	‐	‐	‐	‐
Intermediate risk (scores 1–4/2–6)	83 (78–87)	75 (70–80)	67 (61–73)	50 (42–58)	94 (90–96)	90 (85–94)	89 (84–93)	83 (75–89)	‐	‐	‐	‐
High risk (scores 5–9/7–13)	81 (77–85)	71 (66–76)	60 (53–65)	49 (41–56)	96 (93–98)	93 (89–96)	90 (84–93)	79 (69–87)	‐	‐	‐	‐
Highest risk (scores 10–12/14–23)	75 (41–91)	63 (27–85)	63 (27–85)	63 (27–85)	99 (95–100)	98 (93–99)	92 (84–96)	89 (78–95)	‐	‐	‐	‐
The J‐NICE model (recurrence/progression/cancer‐specific death)												
Low risk (scores 0–4/ 0–2/0–1)	94 (90–96)	91 (87–93)	83 (78–87.1)	70 (62–76)	99 (98–100)	99 (97–100)	97 (95–99)	94 (88–97)	‐	‐	99 (97–100)	98 (93–99)
Intermediate risk (scores 5–6/3–9/4)	84 (77–89)	72 (64–78)	61 (52–69)	45 (32–57)	92 (88–95)	89 (84–93)	85 (79–90)	72 (61–81)	99 (94–100)	96 (90–99)	93 (86–97)	90 (80–95)
High risk (scores 7–16/10–19/5)	63 (55–69)	46 (39–54)	36 (28–44)	24 (14–34)	89 (78–95)	78 (65–87)	73 (59–83)	65 (44–80)	98 (94–99)	96 (91–99)	91 (83–95)	88 (79–94)

Abbreviations: BCG, bacillus Calmette‐Guerin; CI, confidence interval; EAU, European Association of Urology; EORTC, European Organization for Research and Treatment of Cancer; J‐NICE, Japanese NIshinihon uro‐onCology Extensive collaboration group; NMIBC, non‐muscle invasive bladder cancer.

**TABLE 3:** Subgroups of very high‐risk population defined according to the 2021 EAU model.

Subgroup	The BCG cohort	The non‐BCG cohort
Total number of patients with vey high‐risk tumour	731 (100%)	130 (100%)
● Ta HG/G3 and CIS with all 3 risk factors^a^	29 (4.0%)	2 (1.5%)
● T1 G2 and CIS with at least 2 risk factors^a^	40 (5.5%)	3 (2.3%)
● T1 HG/G3 and CIS with at least 1 risk factor^a^	425 (58.1%)	64 (49.2%)
● T1 HG/G3 no CIS with all 3 risk factors^a^	47 (6.4%)	14 (10.8%)
● Either of the following pathologic features	190 (26.0%)	47 (36.2%)
⋄ CIS in the prostatic urethra	48 (6.6%)	0
⋄ Lymphovascular invasion (LVI)	83 (11.4%)	32 (24.6%)
⋄ Micropapillary variant, plasmacytoid variant, sarcomatoid variant or neuroendocrine variant histology	59 (8.1%)^b^	15 (11.5%)^c^

Abbreviations: BCG, bacillus Calmette‐Guerin; CIS, carcinoma in situ; EAU, European Association of Urology.

^a^ Three additional clinical risk factors are age >70 years old, multiple tumour and tumour diameter ≥ 3 cm.

^b^ Four patients had both LVI and variant histology.

^c^ Nine patients had both LVI and variant histology.

### 3.3 Subgroup analysis of the very high‐risk population defined according to the 2021 EAU model

**FIGURE 5:**
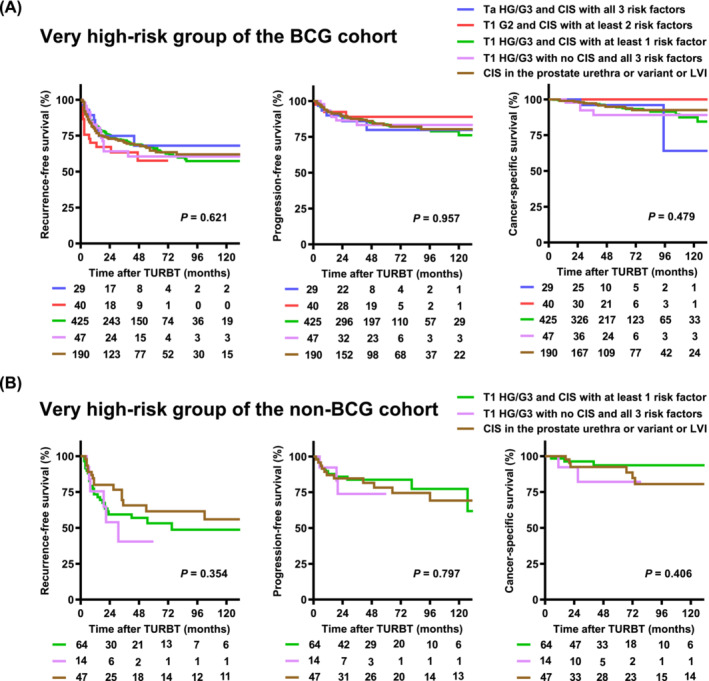
Subgroup survival analysis of very high‐risk group in the 2021 EAU risk model in the BCG (A) and non‐BCG (B) cohorts. The 2021 EAU risk model defines five subgroups as the very high‐risk population based on the T category, WHO 1973 tumour grade, WHO 2004/2016 and three additional clinical factors (Table 1). Survival curves are compared among the five subgroups in the BCG cohort (A). There are only two and three patients with ‘Ta HG/G3 and CIS with all three risk factors’ and ‘T1 G2 and CIS with at least two risk factors’ subgroups, respectively. Thus, survival curves are compared among the remaining three subgroups in the non‐BCG cohort (B). BCG, bacillus Calmette‐Guerin; CIS, carcinoma in situ; HG, high‐grade.

The very high‐risk population consisted of five subgroups based on the T category, WHO 1973 tumour grade, WHO 2004/2016 tumour grade and three additional clinical factors (Table 3). The two most prevalent subgroups among the BCG and non‐BCG cohorts were those with ‘T1 HG/G3 and CIS with at least 1 risk factor’ and ‘CIS in the prostatic urethra, lymphovascular invasion or variant histology’. Survival analysis of the BCG cohort showed no significant differences among the five subgroups (Figure 5A). After excluding two subgroups with fewer than five patients, the survival analysis of the non‐BCG cohort showed no significant differences among the three subgroups (Figure 5B).

## 4 DISCUSSION

The present study clearly demonstrated that the updated 2021 EAU risk model was superior to the 2019 EAU model, particularly concerning progression risk prediction. Much effort has been made to develop a universal risk‐prediction model for the treatment of NMIBC that can aid in the selection of candidates who require intravesical therapy, repeat TURBT or early cystectomy. Consistent results were observed in both the BCG and non‐BCG cohorts. These findings indicate the wide feasibility and applicability of the 2021 EAU risk model for patients with NMIBC. The uniqueness of the 2021 EAU model includes the use of both the WHO 1973 and WHO 2004/2016 grading classifications, which have been recommended in clinical practice for over a decade.^4^, ^15^, ^16^, ^17^, ^18^ Hence, the updated EAU‐NMIBC guideline recommendation to use both the WHO classification systems proved correct.

Lobo et al.^8^ conducted an external validation study of the EAU 2021 progression risk stratification in 529 patients who received iBCG at least with a median follow‐up of 47 months. They found that the 1‐year progression rate in the very high‐risk group of patients receiving at least iBCG was 6.9%, which was equivalent to our result (7.4%); however, both rates were lower than the EAU‐predicted rate (16%). Similar results were observed in the 5‐year progression rates of the very high‐risk group—16.7% for the Lobo et al. cohort, 15.9% for our BCG cohort and 40% for the EAU‐predicted rates. Another study of 1812 patients with high‐risk T1G3 tumours treated with mBCG for 1–3 years reported 1‐ and 5‐year progression rates of 11.4% for and 19.8%, respectively.^19^ These findings strongly suggested that the EAU 2021 risk stratification overestimated the risk of progression in BCG‐treated patients. Additionally, the 1‐ and 5‐year progression rates in the very high‐risk group in the non‐BCG cohort were 11.6% (7.0–18.8) and 21.0% (14.3–30.2), respectively, which were lower than the EAU‐predicted rates (16% and 40%, respectively). A population‐based analysis using linked SEER‐Medicare data demonstrated that the 5‐year progression rate in 7410 patients with HG NMIBC was 22.8%.^19^ Large‐scale studies found approximately half of the 5‐year progression rates (approximately 20%) compared to those predicted by the EAU 2021 (approximately 40%). Evidence from these studies should be used in conjunction with updated risk groups to counsel patients with high‐risk NMIBC regarding the risk of progression with and without BCG. Although the 2021 EAU risk grouping well‐stratified progression risks in the independent validation cohort, the findings suggested that the stratification overestimated progression risks for both patients treated with and without BCG. Possible reason of the discrepancy in risk estimation among the studies might include the difference in patients' background and difference in treatment strategy, such as use of photodynamic diagnosis guidance and response to treatment, such as intravesical therapy using BCG and chemotherapy.

It has been more than 10 years since CUETO and EORTC prediction tools were developed, and modification and recalibration have led to significant improvements in the performance and expansion of their clinical use.^20^, ^21^ Jobczyk et al. provided a free, web‐based calculator that estimates RFS and PFS (https://biostat.umed.pl/deepNMIBC/), developed through a deep learning‐based recalibration of the CUETO and EORTC prediction tools. This risk prediction model uniquely included data on the administration of BCG and mitomycin C, which can help assess the benefits or risks associated with these therapies in patients with specific clinicopathological factors. In this study, original risk‐scoring models were included as comparators. The 2021 EAU model had a better predictive ability for prediction or progression risk than the CUETO and EORTC models in the BCG (C‐index, 0.617 vs. 0.599, *P* = 0.34) and non‐BCG (0.718 vs. 0.541, *P* = 0.006) cohorts. To perform ethnically matched evaluation, we included the J‐NICE risk scoring model, which was developed using a large cohort of Japanese patients in 2020, as a comparator of the 2021 EAU model. The 2021 EAU model had a better predictive ability for prediction or progression risk than the J‐NICE models in the BCG (C‐index, 0.617 vs. 0.540, *P* = 0.028) and non‐BCG (0.718 vs. 0.656, P < 0.006) cohorts. Additionally, the 2021 EAU model does not require a specific calculator, making it easier to use and more user‐friendly.

The improved stratification observed in the 2021 EAU model was attributed to a significant shift in risk grouping from the 2019 EAU model to the 2021 EAU model (Figure 2). In the BCG cohort, a decrease in risk was observed among 246 (8.1%) patients in the high‐to‐intermediate‐risk group and 377 (12.5%) patients in the highest‐to‐high‐risk group. Additionally, 627 (21%) patients showed a decrease in risk, and 264 (8.7%) patients showed an increase in risk, indicating that the 2019 EAU model might overestimate the progression risk in the BCG cohort. Conversely, in the non‐BCG cohort, 190 (24%) and 328 (42%) patients showed a decrease and an increase in risk, respectively, indicating that the 2019 EAU model might underestimate the progression risk.

Moreover, regarding the subgroup analysis of the very high‐risk population defined by the 2021 EAU model, it is mandatory for the NMIBC risk stratification model to accurately identify this small population of very high‐risk patients to consider early cystectomy seriously. The 2019 EAU model recommends early cystectomy for high‐risk patients with any of the following factors: T1 HG/G3 with concurrent bladder CIS, multiple and/or large and/or recurrent T1 HG/G3, T1 HG/G3 with CIS in the prostatic urethra, variant histology of urothelial carcinoma or lymphovascular invasion. We previously sought potential heterogeneity of the 2019 EAU model‐defined highest‐risk patients and found that the subgroup with T1 HG/G3 accompanied with CIS in the prostatic urethra had a worse prognosis compared to the other subgroups.^22^ There was also a significant difference in the clinical behaviour among the five subgroups included in the very high‐risk population (Table S1). There were no significant differences in RFS, PFS or CSS between the subgroups (Figure 5). However, further analysis with a larger sample size is required to confirm the potential heterogeneity.

This study has several limitations. This retrospective study design derived from 31 institutions had an inherent potential for selection bias, inconsistencies in surgical skills and pathological diagnoses, and the decision criteria for adjuvant therapy, timing of changing the treatment and follow‐up schedule were dependent on the discretion of the physician. There was a possibility of errors occurring during data extraction from hospital medical charts; therefore, stringent data clearance and quality control were performed to reduce the risk of potential bias. Two cohorts, the BCG and non‐BCG cohorts, were used to validate this study. The non‐BCG cohort included patients who did not receive adjuvant treatment and those who underwent repeated intravesical chemotherapy. To prepare the non‐BCG cohort, 561 patients who received intravesical BCG therapy were excluded from the analysis. This patient selection could cause a significant bias, especially in the high‐risk/highest risk patients for whom BCG treatment is the recommended treatment according to current guidelines. The reasons for guideline non‐adherence were not recorded in this study. Advances in treatment strategies, diagnostic techniques and TURBT modality may have the potential to improve outcomes and reduce the risk of progression in contemporary patients. The statistical power may be limited because of the small number of patients and events in some subgroups. Finally, the present study did not include comparison with risk stratification models released from National Comprehensive Cancer Network and American Urological Association. Our focus was to compare two risk models provided from EAU guidelines and score calculation‐based risk models, such as EORTC, CUETO and J‐NICE models.

In conclusion, the updated 2021 EAU risk model for the treatment of NMIBC has the potential to address the limitations of the current risk models. This user‐friendly and straightforward prediction tool can assist clinicians in determining the most appropriate adjuvant treatment and identifying patients who may require intensified surveillance or early cystectomy. Further external validation with more extensive and diversified cohorts is vital to enhance its real‐world clinical impact.

## AUTHOR CONTRIBUTIONS

Makito Miyake, the first author, made significant contributions to the data collection and interpretation and writing of the original manuscript. Hiroshi Kitamura made significant contribution to the research design. Naotaka Nishiyama, Tatsuki Miyamoto, Tomonori Nakahama, and Tomomi Fujii performed the formal analysis. Hiroaki Matsumoto, Hideyasu Matsuyama, Masaya Yonemori, Hideki Enokida, Rikiya Taoka, Takashi Kobayashi, Takahiro Kojima, and Yoshiyuki Matsui performed the data acquisition. Naotaka Nishiyama, Hiroyuki Nishiyama, and Kiyohide Fujimoto were involved in interpretation of the data and contributed important intellectual input in manuscript writing—review and editing. All of the authors meet criteria for authorship, have read the manuscript, and have approved this submission.

## CONFLICT OF INTEREST STATEMENT

The authors disclose no potential conflict of interest.

## Supporting information


**Table S1.** Clinical composition of the former and current EAU NMIBC prognostic factor risk groups based prognostic factor risk groups based on the WHO1973 and WHO2004/2016 grading systems.

**Table S2.** The EORTC, CUETO, and J‐NICE risk tables for calculating recurrence, progression, and cancer‐specific death scores.

**Table S3.** Clinicopathologic variables and comparison according to the 2021 EAU risk stratification in the BCG cohort.

**Table S4.** Clinicopathologic variables and comparison according to the 2021 EAU risk stratification in the non‐BCG cohort.

**Data S1.** Supporting Information
