# Supplementary material for: Validation of non‐muscle‐invasive bladder cancer risk stratification updated in the 2021 European Association of Urology guidelines
Source: BJUI Compass. 2023 Nov 3;5(2):269–80. doi: 10.1002/bco2.305 (PMC10869660; doi:10.1002/bco2.305)
Supplement: Supplementary file 1 — Table S1. Clinical composition of the former and current EAU NMIBC prognostic factor risk groups based prognostic factor risk groups based on the WHO1973 and WHO2004/2016 grading systems. [file BCO2-5-269-s002.pdf]

Table S1. Clinical composition of the former and current EAU NMIBC prognostic factor risk groups based on the WHO1973 and WHO2004/2016 grading systems

Risk group	The 2019 EAU NMIBC prognostic factor risk groups	The 2021 EAU NMIBC prognostic factor risk groups based on the WHO1973 and WHO2004/2016 grading systems
Low risk	Primary, solitary, TaG1 (PUNLMP, LG), <3 cm, no CIS	● A primary, single, Ta LG/G1 tumor ≤3 cm in diameter without CIS in a patient ≤70 yo<br>● A primary LG/G1 tumor with at most one of the following additional clinical risk factors:<br>◊ Age > 70 yo<br>◊ Multiple tumor<br>◊ Tumor diameter ≥ 3 cm<br>◊ T1 tumor
Intermediate risk	All tumours not defined in the other categories (between the category of low and high risk)	Patients without CIS who are not included in either the low-, high-, or very high-risk groups
High risk	Any of the following:<br>● T1 tumour<br>● HG/G3 tumour<br>● CIS<br>● Multiple, recurrent, and large (>3 cm) Ta LG/G1-2 tumours (all features must be present)	● All T1 HG/G3 without CIS, exept those included in the very high-risk group<br>Stage, grade with additional clinical risk factors:<br>● Ta LG/G2 or T1 G1, no CIS with all 3 risk factors<br>● Ta HG/G3 or T1 LG, no CIS with at least 2 risk factors<br>● T1 G2, no CIS with at least 1 risk factor<br>◊ Age > 70 yo<br>◊ Multiple tumor<br>◊ Tumor diameter ≥ 3 cm
Highest risk in the 2019 EAU model<br>or<br>Vey high risk in the 2021 EAU model	Subgroup in the high-risk group<br>Any of the following:<br>● T1 HG/G3 with concurrent bladder CIS<br>● Multiple and/or large and/or recurrent T1 HG/G3<br>● T1 HG/G3 with CIS in the prostatic urethra<br>● Some forms of variant histology of urothelial carcinoma<br>● Lymphovascular invasion	Stage, grade with additional clinical risk factors:<br>● Ta HG/G3 and CIS with all 3 risk factors<br>● T1 G2 and CIS with at least 2 risk factors<br>● T1 HG/G3 and CIS with at least 1 risk factor<br>● T1 HG/G3 no CIS with all 3 risk factors<br>◊ Age > 70 yo<br>◊ Multiple tumor<br>◊ Tumor diameter ≥ 3 cm<br>● Patients with CIS in the prostatic urethra, lymphovascular invasion, and micropapillary, plasmacytoid, sarcomatoid, or neuroendocrine variant histology should be included.

CIS, carcinoma in situ; EAU, European Association of Urology; HG, high grade; LG, low grade; NMIBC, non-muscle invasive bladder cancer; WHO, World Health Organization
