# Supplementary material for: Validation of non‐muscle‐invasive bladder cancer risk stratification updated in the 2021 European Association of Urology guidelines
Source: BJUI Compass. 2023 Nov 3;5(2):269–80. doi: 10.1002/bco2.305 (PMC10869660; doi:10.1002/bco2.305)
Supplement: Supplementary file 2 — Table S2. The EORTC, CUETO, and J‐NICE risk tables for calculating recurrence, progression, and cancer‐specific death scores. [file BCO2-5-269-s001.pdf]

Table S2. The EORTC, CUETO, and J-NICE risk tables for calculating recurrence, progression, and cancer-specific death scores

Factor	The EORTC risk table		The CUETO risk table		The J-NICE risk table		
	Recurrence (6 factors)	Progression (6 factors)	Recurrence (6 factors)	Progression (6 factors)	Recurrence (6 factors)	Progression (5 factors)	Cancer-specific death (2 factors)
Tumor multifocality							
Single	0	0	0	0	0	0	-
Multifocal (2–3)	3	3	0	0	2	2	-
Multifocal (4–7)	3	3	2	1	2	2	-
Multifocal (≥ 8)	6	3	2	1	2	2	-
Tumor size							
< 3 cm	0	0	-	-	0	0	-
≥ 3 cm	3	3	-	-	2	2	-
Prior recurrence history							
Primary case	0	0	0	0	0	0	-
Recurrent case (0–1/year)	2	2	4	2	6	6	-
Recurrent case (≥ 2/year)	4	2	4	2	6	6	-
T category							
Ta	0	0	-	0	3	-	-
T1	1	4	-	2	0	-	-
Carcinoma in situ							
No	0	0	0	0	-	-	-
Yes	1	6	2	1	-	-	-
Tumor grade (WHO1973)							
G1	0	0	0	0	-	-	-
G2	1	0	1	2	-	-	-
G3	2	5	3	6	-	-	-
Tumor grade (WHO2004/2016)							
Low-grade	-	-	-	-	0	0	0
High-grade	-	-	-	-	2	7	4
Age							
< 60 yo	-	-	0	0	0	-	0
60–70 yo	-	-	1	0	0	-	0
> 70 yo	-	-	2	2	1	-	1
Sex							
Male	-	-	0	-	-	0	-
Female	-	-	3	-	-	2	-
Total scores	0–17	0–23	0–16	0–14	0–16	0–19	0–5
Risk stratification							
Low risk	0	0	0-4	0-4	0–4	0–2	0–1
Intermediate risk	1–4	2–6	5–6	5–6	5–6	3–9	4
High risk	5–9	7–13	7–9	7–9	7–16	10–19	5
Highest risk	10–17	14–23	10–16	10–14	–	–	–

EORTC, European Organization for Research and Treatment of Cancer; CUETO, Club Urologico Espanol de Tratamiento Oncologico; J-NICE, Japanese NIshinihon uro-onCology Extensive collaboration group; WHO, the World Health Organization
