# Supplementary material for: Validation of non‐muscle‐invasive bladder cancer risk stratification updated in the 2021 European Association of Urology guidelines
Source: BJUI Compass. 2023 Nov 3;5(2):269–80. doi: 10.1002/bco2.305 (PMC10869660; doi:10.1002/bco2.305)
Supplement: Supplementary file 3 — Table S3. Clinicopathologic variables and comparison according to the 2021 EAU risk stratification in the BCG cohort. [file BCO2-5-269-s003.pdf]

Table S3. Clinicopathologic variables and comparison according to the 2021 EAU risk stratification in the BCG cohort

Variables		Total	The 2021 EAU risk grouping			
			Low risk	Intermediate risk	High risk	Very high risk
N		3024 (100%)	0	548 (18%)	1745 (58%)	731 (24%)
Age at TURBT	Median (interquartile range)	72 (65–78)	-	69 (63–76)	72 (66–78)	73 (66–79)
Sex	Male	2,497 (82.6%)	-	451 (82.3%)	1,431 (82.0%)	615 (84.1%)
	Female	527 (17.4%)	-	97 (17.7%)	314 (18.0%)	116 (15.8%)
Prior recurrence history	Primary case	2,273 (75.2%)	-	270 (49.3%)	1,344 (77.0%)	659 (90.2%)
	Recurrent case (0–1/year)	751 (24.8%)	-	278 (50.7%)	401 (23.0%)	72 (9.8%)
Tumor multifocality	Single	1871 (61.9%)	-	321 (58.6%)	1178 (67.5%)	372 (50.9%)
	Multifocal (2–3)	774 (25.6%)	-	131 (23.9%)	407 (23.3%)	236 (32.3%)
	Multifocal (4≤)	379 (12.5%)	-	96 (17.5%)	160 (9.2%)	123 (16.8%)
Tumor size	<3 cm	1,862 (61.6%)	-	411 (75.0%)	1,053 (60.3%)	398 (54.4%)
	≥3 cm	505 (16.7%)	-	56 (10.2%)	221 (12.7%)	228 (31.2%)
	Unknown	657 (21.7%)	-	81 (14.8%)	471 (27.0%)	105 (14.4%)
T category	Ta	925 (30.6%)	-	511 (93.2%)	368 (21.1%)	46 (6.3%)
	T1	1,495 (49.4%)	-	37 (6.8%)	804 (46.1%)	654 (89.5%)
	Tis	604 (20.0%)	-	0 (0.0%)	573 (32.8%)	31 (4.2%)
Tumor grade (WHO1973)	G1	79 (2.6%)	-	65 (11.9%)	14 (0.8%)	0 (0.0%)
	G2	939 (31.1%)	-	367 (67.0%)	479 (27.4%)	93 (12.7%)
	G3	2,006 (66.3%)	-	116 (21.2%)	1,252 (71.7%)	638 (87.3%)
Tumor grade (WHO2004/2016)	Low-grade	330 (10.9%)	-	273 (49.8%)	55 (3.2%)	2 (0.3%)
	High-grade	2,694 (89.1%)	-	275 (50.2%)	1,690 (96.8%)	729 (99.7%)
Concurrent carcinoma in situ	No	2,122 (70.2%)	-	548 (100.0%)	1,350 (77.4%)	224 (30.6%)
	Yes	902 (29.8%)	-	0 (0.0%)	395 (22.6%)	507 (69.4%)
Non-urothelial carcinoma variant histology	No	2,922 (96.6%)	-	548 (100.0%)	1,745 (100.0%)	629 (86.0%)
	Divergent differentiation	73 (2.4%)	-	0 (0.0%)	0 (0.0%)	73 (10.0%)
	Variant morphologies	29 (1.0%)	-	0 (0.0%)	0 (0.0%)	29 (4.0%)
Lymphovascular invasion	No	2,886 (95.4%)	-	548 (100.0%)	1,745 (100.0%)	593 (81.1%)
	Yes	138 (4.6%)	-	0 (0.0%)	0 (0.0%)	138 (18.9%)
Restaging/repeat TUR	No	2005 (66.3%)	-	462 (85.3%)	1240 (71.1%)	303 (41.5%)
	Performed	1019 (33.7%)	-	86 (15.7%)	505 (28.9%)	428 (58.5%)
Immediate postoperative intravesical chemotherapy	No	2183 (72.2%)	-	350 (63.8%)	1268 (72.5%)	566 (77.3%)
	Performed	841 (27.8%)	-	198 (36.1%)	477 (27.3%)	166 (22.7%)
Maintenance BCG followed by induction BCG	No	2508 (82.9%)	-	419 (76.5%)	1456 (83.4%)	633 (86.6%)
	Performed	516 (17.1%)	-	129 (23.5%)	289 (16.6%)	98 (13.4%)

EAU, European Association of Urology; TURBT, transurethral resection of the bladder tumor; WHO, the World Health Organization; BCG, bacillus Calmette-Guerin
