# Supplementary material for: Validation of non‐muscle‐invasive bladder cancer risk stratification updated in the 2021 European Association of Urology guidelines
Source: BJUI Compass. 2023 Nov 3;5(2):269–80. doi: 10.1002/bco2.305 (PMC10869660; doi:10.1002/bco2.305)
Supplement: Supplementary file 4 — Table S4. Clinicopathologic variables and comparison according to the 2021 EAU risk stratification in the non‐BCG cohort. [file BCO2-5-269-s004.pdf]

Table S4. Clinicopathologic variables and comparison according to the 2021 EAU risk stratification in the non-BCG cohort

Variables		Total	The 2021 EAU risk grouping			
			Low risk	Intermediate risk	High risk	Highest risk
N		789	173 (22%)	269 (34%)	217 (28%)	130 (16%)
Age at TURBT	Median (interquartile range)	71 (63–78)	65 (59–73)	71 (62–78)	73 (66–80)	72 (67–78)
Sex	Male	663 (84.0%)	142 (82.1%)	229 (85.1%)	183 (84.3%)	109 (83.8%)
	Female	126 (16.0%)	31 (17.9%)	40 (14.9%)	34 (15.7%)	21 (16.1%)
Prior recurrence history	Primary case	619 (78.5%)	135 (78.0%)	202 (75.0%)	169 (78.0%)	113 (86.9%)
	Recurrent case (0–1/year)	170 (21.5%)	38 (22.0%)	67 (25.0%)	48 (22.0%)	17 (13.1%)
Tumor Multifocal	Single	464 (59.1%)	144 (83.2%)	168 (62.4%)	102 (47.0%)	52 (40.0%)
	Multifocal (2–8)	323 (40.9%)	29 (16.8%)	101 (37.6%)	115 (53.0%)	78 (60.0%)
Tumor size	<3 cm	663 (84.0%)	168 (97.1%)	241 (89.6%)	169 (77.9%)	85 (65.4%)
	≥3 cm	126 (16.0%)	5 (2.9%)	28 (10.4%)	48 (22.1%)	45 (34.6%)
T category	Ta	449 (56.9%)	169 (97.7%)	229 (85.1%)	47 (21.7%)	4 (3.1%)
	T1	331 (42.0%)	4 (2.3%)	40 (14.9%)	161 (74.2%)	126 (96.9%)
	Tis	9 (1.1%)	0 (0.0%)	0 (0.0%)	9 (4.1%)	0 (0.0%)
Tumor grade (WHO1973)	G1	196 (24.9%)	152 (87.9%)	43 (16.0%)	128 (59.0%)	1 (0.7%)
	G2	378 (47.9%)	0 (0.0%)	211 (78.4%)	78 (35.9%)	39 (30.0%)
	G3	176 (22.3%)	0 (0.0%)	9 (3.3%)	11 (5.1%)	89 (68.6%)
	Undefined	39 (4.9%)	21 (12.1%)	6 (2.3%)	0 (0.0%)	1 (0.7%)
Tumor grade (WHO2004/2016)	Low-grade	493 (62.5%)	173 (100%)	243 (90.3%)	75 (34.6%)	2 (1.5%)
	High-grade	296 (37.5%)	0 (0.0%)	26 (9.7%)	142 (65.4%)	128 (98.5%)
Concurrent carcinoma in situ	No	686 (87.0%)	173 (100%)	269 (100%)	184 (84.8%)	60 (46.2%)
	Yes	103 (13.0%)	0 (0.0%)	0 (0.0%)	33 (15.2%)	70 (53.8%)
Non-urothelial carcinoma variant histology	No	765 (97.0%)	173 (100%)	269 (100.0%)	217 (100.0%)	106 (81.5%)
	Divergent differentiation	11 (1.4%)	0 (0.0%)	0 (0.0%)	0 (0.0%)	11 (8.5%)
	Variant morphologies	13 (1.6%)	0 (0.0%)	0 (0.0%)	0 (0.0%)	13 (10.0%)
Lymphovascular invasion	No	709 (89.9%)	173 (100%)	269 (100%)	210 (96.8%)	57 (43.8%)
	Yes	63 (8.0%)	0 (0.0%)	0 (0.0%)	0 (0.0%)	63 (48.5%)
	Unknown	17 (2.1%)	0 (0.0%)	0 (0.0%)	7 (3.2%)	10 (7.7%)
Restaging/repeat TUR	No	751 (95.2%)	172 (99.9%)	265 (98.5%)	202 (93.1%)	113 (86.9%)
	Performed	38 (4.8%)	1 (0.1%)	4 (1.5%)	15 (6.9%)	17 (13.1%)
Immediate postoperative intravesical chemotherapy	No	623 (72.0%)	149 (86.1%)	212 (78.8%)	160 (73.7%)	104 (80.0%)
	Performed	166 (21.0%)	24 (13.9%)	57 (21.2%)	57 (26.3%)	26 (20.0%)
Maintenance intravesical chemotherapy	No	769 (81.3%)	154 (89.0%)	220 (81.8%)	164 (75.6%)	107 (82.3%)
	Performed	144 (18.7%)	19 (11.0%)	49 (18.2%)	53 (24.4%)	23 (17.7%)

EAU, European Association of Urology; TURBT, transurethral resection of the bladder tumor; WHO, the World Health Organization; BCG, bacillus Calmette-Guerin
