# Supplementary figures and images for: Validation of non‐muscle‐invasive bladder cancer risk stratification updated in the 2021 European Association of Urology guidelines
Source: BJUI Compass. 2023 Nov 3;5(2):269–80. doi: 10.1002/bco2.305 (PMC10869660; doi:10.1002/bco2.305)

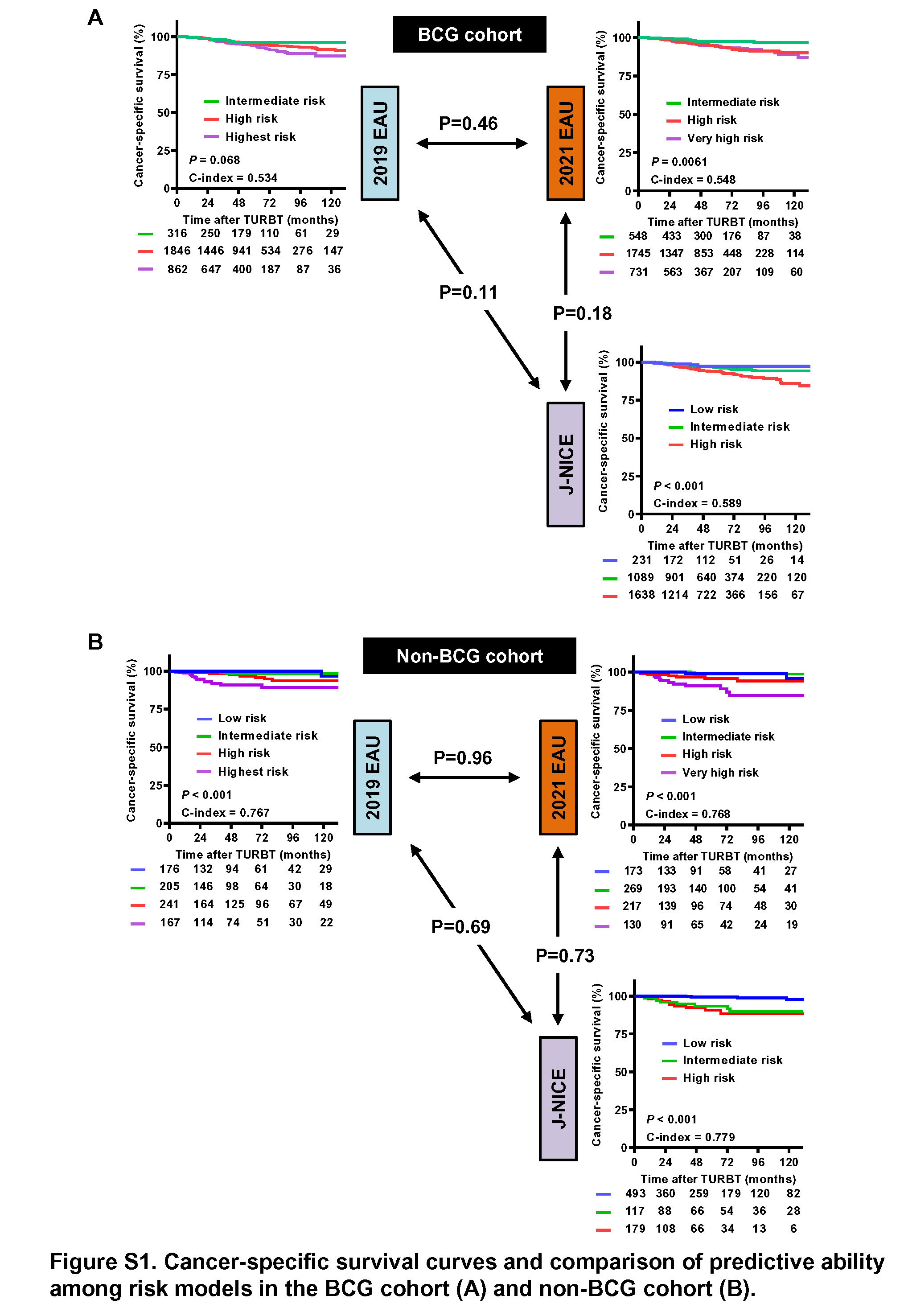

Supplement: Supplementary file 5 — Data S1. Supporting Information [file BCO2-5-269-s005.tif]
